# Enzymatic digestion improves testicular sperm retrieval in non-obstructive azoospermic patients

**Published:** 2013-06

**Authors:** Tahereh Modarresi, Marjan Sabbaghian, Abdolhossein Shahverdi, Hani Hosseinifar, Ali Asghar Akhlaghi, Mohammad Ali Sadighi Gilani

**Affiliations:** 1 *Department of Andrology at Reproductive Biomedicine Research Center, Royan Institute for Reproductive Biomedicine, ACECR, Tehran, Iran.*; 2 *Department of Embryology at Reproductive Biomedicine Research Center, Royan Institute for Reproductive Biomedicine, ACECR, Tehran, Iran.*; 3 *Department of Epidemiology and Reproductive Health at Reproductive Biomedicine Research Center, Royan Institute for Reproductive Biomedicine, ACECR, Tehran, Iran.*; 4 *Department of Urology, Shariati Hospital, Tehran University of Medical Sciences, Tehran, Iran.*

**Keywords:** *TESE*, *Enzymatic digestion*, *Non-obstructive azoospermia*, *FSH*, *LH*

## Abstract

**Background: **In patients with non-obstructive azoospermia (NOA), vital spermatozoa from the tissue is obtained from testes by enzymatic treatment besides the mechanical treatment.

**Objective:** To increase the sperm recovery success of testicular sperm extraction (TESE), with enzymatic digestion if no sperm is obtained from testis tissue by mechanical method.

**Materials and Methods: **Tissue samples were collected from 150 men who presented with clinical and laboratory data indicating NOA by means of TESE and micro dissection TESE methods. Initially, mature spermatozoa were examined for by mechanical extraction technique shredding the biopsy fractions. In cases whom no spermatozoa was observed after maximum 30 min of initial searching under the inverted microscope, the procedure was followed by enzymatic digestion using DNaseI and collagenase type IV. Surgery type, pathology, AZF, karyotype, hormones and testis size were compared in patients.

**Results:** Of 150 cases with NOA, conventional mincing method extended with enzymatic treatment yielded successful sperm recovery in 13 (about 9%) patients. Comparison of parameters revealed that level of FSH and LH were significantly different (p=0.04 and 0.08 respectively) between two groups that response negative and positive to enzymatic digestion.

**Conclusion:** The combination of conventional TESE and enzymatic digestion is an effective method to recover spermatozoa. The benefit of the mincing combined with enzyme to sperm retrieval for NOA firstly shorten the mechanical searching time, leading to minimizing further cellular damage as well as exposure to external conditions, and secondly reduce the number of cases with sperm recovery failures. Also, the serum level of FSH and LH are factors that influence the chance of sperm retrieval.

## Introduction

Testicular sperm retrieval combined with intracytoplasmic sperm injection (ICSI) has been the first-line treatment in non-obstructive azoospermia (NOA) (1). When the ejaculate contains insufficient vital spermatozoa, ICSI can also be performed (2, 3). In cases of several testicular failures or on successful epididymal sperm retrieval, however, the testis is the only source of spermatozoa. Several pregnancies have been obtained after ICSI with testicular spermatozoa (3, 4). Several studies demonstrated that open biopsies for testicular sperm extraction (TESE) are required to achieve an optimal chance of finding the rare foci of spermatozoa present within the functionally impaired gonads (5, 6). In TESE suitable sperm for injection from testicular tissue can be obtained after mechanical preparation by mincing and shredding the whole tissue (7). 

Patients with hypospermatogenesis have an invariably high sperm recovery rate by the use of mechanical TESE procedure; there is failure to obtain spermatozoa for ICSI in 25-50% of men with more severe testis failure (8, 9). In order to find the rare spermatozoa present in the testes of men with limited sperm production, either extensive multiple biopsies from every area of the testis should be performed or large amounts of testicular tissue should be removed. Schlegel introduced concept of microdissection TESE, which, besides minimizing the tissue excision, could improve sperm yield from men with NOA (10). 

Although this technique is safer than standard open surgical TESE, failure to find spermatozoa may still occur in up to 53% of NOA patients (11). Mechanical separation also causes contamination of testicular tissue suspension with many damaged cells and residual tissue pieces. Whether rough extraction method during TESE induces significant reactive oxygen species (ROS) released by damaged cells and impairs sperm function needs to be investigated. Besides the mechanical treatment, vital spermatozoa from the tissue obtained from testis by biopsy have been enzymatically prepared (12). Isolated human testicular germ cells were extracted from enzymatic digestion of testicular tissue using collagenase type IA or trypsin-DNase method (13, 14). The first pregnancy after ICSI using spermatozoa extracted by this method has been reported by Fisher *et al* (15). However, incubation with collagenase type IA required a minimum of 4h to disperse the testicular tissue, with a resultant decrease in motility (12, 14). 

Collagenase type IV has been found to be more efficient than collagenase type I for testicular sperm recovery (12). Although collagenase type IV requires shorter incubation time, enzymatic digestion has been reported to lead to the formation of intercellular bridges in the mammalian testis, which connect the developing germ cells together. Thus, enzymatic method alone is not able to disperse germ cells completely from seminiferous epithelium. To overcome these drawbacks, Crabbe *et al* suggested an alternative method combining the mechanical mincing of the testicular tissue with the enzymatic treatment (16). 

In this present study, we investigated the sperm recovery success of conventional mincing method extended with enzymatic treatment for microsurgically obtained testis tissue in NOA cases.

## Materials and methods

For this case-control study, testicular tissue (n=150) was obtained from surgical testis biopsies from non-obstructive azoospermic patients referred to Royan Institute between 2011 and 2012.The study was accepted by the Institutional Review Board of the Royan Institute and participants provided written informed acceptance, permitting the use of their tissue samples. 

All samples which sperm exctraction were failed by mechanical extraction technique the biopsy fraction in embryology lab, were subjected to enzymatically digestion method, and then were observed by inverted microscope. The surgical techniques for testicular biopsy are conventional TESE and microscopic TESE. For TESE, after sedation and local anasthesia a small tissue sample (testicular biopsy) was taken through a small incision in the scrotum and testis. Each sample was placed in a petri dish filled with 1ml Ham's F10 medium and was mechanically cut and dispersed by an embrologist. 

A small droplet of dispersed tissue suspension was replaced on the slide glass and examined under an inverted microscope at 400× magnification. If no spermatozoa were seen, subsequent samples were taken. If no sperm was seen in the operating room, all testicular samples were subjected to centrifugation a 3000 r.p.m with 5ml Ham's F10 and examined to determine the presence of even a single sperm. Microsurgical TESE was performed under local anasthesia with examination of the seminoferous tubules using an operating microscope. Enlarged seminoferous tubules were removed and evaluated by an embryologist as mentioned above. 

The age of patients undergoing TESE and microscopic TESE ranged from 23-50 years (average 33.8 year). During the testicular intervention, one small tissue specimen obtained surgically was placed in Bouin̒s solution and sent for histology examination. Testicular histology was classified into Sertoli cell only (the absence of germ cells in the seminiferous tubules), maturation arrest (an interruption in the development of spermatogonia to mature sperm at the level of spermatogonia, spermatocytes or spermatids) and complete tubular hyalinization (the tubules were filled with collagen fibers) (17, 18). After biopsy, all testicular tissue pieces were transferred to a petridish filled with Ham's F10 solution, and mature spermatozoa were searched for by mechanical extraction shredding the biopsy fractions using two fine needles. Microscopic examination of the suspension was carried out under the inverted microscope. If spermatozoa were found, they were frozen and the next step was ICSI. However, in cases that no sperm was observed after maximum 30 min of initial searching, the procedure was followed by enzymatic digestion. During the enzymatic digestion the tissue suspension containing no spermatozoa after mechanical extraction was transferred to a conical tube in the incubation medium containing 25 µg/ml DNAse (Sigma DN25) and 1000 IU/ml of collagenase type IV (Sigma C5138) (12, 16). 

After incubation at 37^o^C for 1h, the resultant solution was centrifuged for 5 min at 50g to remove remaining tissue residues. Diluting the supernatant with fresh medium, two more washing steps were conducted sequentially. Then, the final pellet was re-suspended in the medium and the presence of free spermatozoa was checked under the inverted microscope.

**Statistical analysis**

Statistical evaluation was performed using t-test, chi-square and fisher's exact test. Mean (±SD) were reported for descriptive analysis. SPSS software version 16 was used for statistical analysis. Statistical significant was set at p<0.05.

## Results

Enzymatic digestion of testicular tissue remnants was carried out in 150 patients undergoing TESE and microscopic TESE that no spermatozoa has been found after at least a 30 min search of the shredded biopsy suspensions by mechanical method. Surgical analysis revealed that of the 13 cases whose sperm was found by enzymatic digestion, 1 patient had surgery by TESE and 12 patients had surgery by microscopic TESE (Table I). 

Histological analysis of diagnostic testicular biopsies revealed that of the 13 cases whose sperm was found, 6 patients had Sertoli cell-only syndrome (SCOS) and 7 patients had complete maturation arrest. None of the patients with complete tubular hyalinization, answered positive to enzymatic digestion (Table I). Genetic information of the patients were exctracted from their records. Karyotype was studied in 78 patients that 66 patients had normal karyotype and 12 patients had klinefelter syndrome. In 66 patients, 6 (9.1%) patients had positive response to enzymatic digestion. 

In none of the patients with klinefelter syndrome, sperm was found by enzymatic digestion (Table I). Of 51 patients with normal AZF, 4 patients (7.8%) answered positive to enzymatic digestion and of 4 patients with AZFc deletion, no patient (0%) answered positive to enzymatic digestion (Table I). Hormonal analysis of patients is shown in Table II. The mean of the level of LH was 3.8±2.34 in group that sperm was found (8 cases) and it was 7.67±6 in group that sperm was not found (75 cases) by enzymatic digestion. The mean level of FSH hormone in group that responded negative was 22.76±12.84 and in group that responded positive with 8 patients was 10.76±8.65. 

The mean of the level of testosterone and prolactin were not significantly different between two groups. The mean age of the men with positive results was relatively lower than those with negative results in enzymatic digestion (30.85±5.1 vs. 34±6.3 years, respectively; p=0.07) (Table II). The mean testis size of the men with positive results was relatively higher than those with negative results in enzymatic digestion (right testis: 4.18±1.6 vs. 3.46±1.9 ml and left testis: 4.50±1.7 vs. 3.51±2.1 ml) that was not statistically significant (Table II).

**Table I:** Pathology, karyotype, AZF and surgery type percent in patients that responded positive and negative to enzymatic digestion

	**Group **	
Pathology	Negative sperm retrieval	Positive sperm retrieval
Sertoli only syndrome	83 (93.3%)	6 (6.7%)
Complete maturation arrest	29 (80.6%)	7 (19.4%)
Complete tubular hyalinization	7 (100%)	0 (0%)
Normal karyotype	60 (90.9%)	6 (9.1%)
Klinefelter syndrome	12 (100%)	0 (0%)
Normal AZF	47 (92.2%)	4 (7.8%)
Microdeletion in AZFc	4 (100%)	0 (0%)
TESE	20 (95.2%)	1 (4.8%)
Micro-TESE	117 (90.7%)	12 (9.3%)

Note: Values are Count (%)

**Table II:** The mean of age and LH, FSH, testosterone, prolactin hormones and testis size in two groups that responded positive and negative to enzymatic digestion

	**Group**	
Characteristic	Negative sperm retrieval	Positive sperm retrieval
Age	34.12 (6.32)	30.85 (5.113)
Prolactin	193.44 (105.914)	177.50 (76.748)
LH (mIU/ml)^*^	7.67 (6.058)	3.80 (2.34)
FSH (mIU/ml)^*^	22.76 (15.842)	10.76 (8.657)
Testostrone (ng/ml)	4.07 (2.589)	3.69 (1.233)
Right testis size	3.46 (1.944)	4.18 (1.602)
Left testis size	3.51 (2.140)	4.50 (1.707)

Note: Values are means ± (SD)

Statistical evaluation was performed using t-test, chi-square test.

## Discussion

In the majority of the patients with testicular failure a few spermatozoa can be extracted. The optimal method of obtaining suitable sperm from testicular tissue is mechanical preparation by mincing and shredding the whole tissue (9). However, enzymatic digestion using DNase and collagenase to loosen the cellular contacts in the tubular walls facilitating release of gametes has also been suggested by others (12, 14). 

The preparations with collagenase type IV was reported to provide high yield of vital testicular spermatozoa, with no increased risk of alteration in cell membrane composition (12). Collagenase type IV probably is the best protease to dissociate the testicular tissue because of type IV collagenase is one of the products secreted by the Sertoli cells and its secretion may play a role in the translocation of germ cells and spermiation, i.e. the release of mature spermatozoa into the lumen of tubules (16).

Different effects of various temperatures for spermatozoa undergoing developmental progression seem to be related with incubation time, as 37^o^C in short-term culture conditions has been shown to be efficient for spermatozoa survival (16). Regarding these data, we tried to shorten the staying time of the tissue suspension at room temperature to avoid the adverse effects of open air conditions on cellular metabolism. Instead of prolonged mechanical mincing work, dissolution of the tissue was accomplished by leaving it in an incubator at 37^o^C for only 1h. Enzymatic digestion alone may not be sufficient to effective dispersal of attached spermatozoa from the seminiferous tubule, and prolonged incubation with enzyme may be required (14). Although collagenase IV and DNAase used in this study have been shown to provide complete dissolution of the cells from their tissue, with a higher yield and higher percentage viability than other enzymatic preparations. 

In the study of Crabbe *et al* in 30% of patients whose no spermatozoa were found after search of the supernatant of the minced suspension, spermatozoa were found after enzymatic treatment of the residual tissue pieces (16). Aydos *et al* reported a sperm retrieval rate of 33% after chemical digestion, for patients whose sperm was not discovered intraoperatively (19). In the present study, a group of men with no spermatozoa had been found after mechanical mincing of testicular tissue pieces underwent enzymatic digestion procedure subsequently, with the hope of finding at least one spermatozoon. In fact, this approach detected spermatozoa in 8.6% of all NOA cases that was similar to the recently report of Ramasamy *et al* (20). 

The higher sperm retrieval in the work of Crabbe *et al* and Aydos *et al* could be related to less efficient initial mechanical mincing than current procedure. It was shown that embryologists' experience had a significant effect on sperm retrieval in laboratory. The sperm was identified in 10 of the 75 patients in the beginning of our study, while it was 3 out of the next 75 patients. It is suggested that there was a learning curve for our embryologist that affect the chance of sperm detection in the laboratory.

Despite of Ramasamy's report, there was not any relationship between different pathology status and the chance of finding sperm in the lab. In the other word, the chance of sperm finding was similar between hypospermatogenesis and maturation arrest and Sertoli-cell only groups. This is maybe related to a smaller number of patients that were studied in the present work. However, there was a significant relationship between serum level of FSH and LH with the chance of sperm retrieval in the lab. This data demonstrated that hormonal level of FSH and LH are identifiable factors influencing the chance of sperm finding in the lab. 
